# Non-steroidal anti-inflammatory drugs and the risk of a second hip fracture: a propensity-score matching study

**DOI:** 10.1186/s12891-016-1047-2

**Published:** 2016-05-04

**Authors:** Po-Yao Chuang, Shih-Hsun Shen, Tien-Yu Yang, Tsan-Wen Huang, Kuo-Chin Huang

**Affiliations:** Department of Orthopaedic Surgery, Chang Gung Memorial Hospital, Chiayi, Taiwan; College of Medicine, Chang Gung University, Taoyuan, Taiwan

**Keywords:** Non-steroidal anti-inflammatory drugs, Fragility hip fracture, Second hip fracture, Population-based study, Propensity-score matching

## Abstract

**Background:**

Non-steroidal anti-inflammatory drugs (NSAIDs) are frequently prescribed for elderly patients, particularly after a hip fracture. However, we are not clear about the effect of NSAIDs on the risk of a second hip fracture because of confounding factors.

**Methods:**

This was a Taiwan National Health Insurance Research Database-based study using propensity-score matching (PSM) to control for confounding. Enrollees were selected from patients with a hip fracture during 1996–2004 and followed longitudinally until December 2009. After PSM for comorbidities and bisphosphonate therapy, 94 patients with a second hip fracture were assigned to the Cases group and 461 without it to the Controls group. The target drugs are NSAIDs; paracetamol and dexamethasone are used for comparison.

**Results:**

The correlation between the mean daily-dose (MDD) ratios of NSAIDs and the probability values of the current statistical tests were highly negative (Pearson’s *r* = −0.920, *P* = 0.003), which indicated that the higher the MDD ratios, the greater the risks of a second hip fracture. A Kaplan-Meier survival analysis showed a time-dependent trend of increasing risk of a second hip fracture in patients taking NSAIDs (*P* < 0.001). Moreover, patients ≥60 years old had a higher risk of a second hip fracture than did those <60 and taking the NSAIDs diclofenac (*P* = 0.016) and celecoxib (*P* = 0.003) and the corticosteroid dexamethasone (*P* = 0.018), but not those taking analgesic paracetamol (*P* = 0.074).

**Conclusions:**

We conclude that taking NSAIDs after a fragility hip fracture dose- and time-dependently significantly increases the risk of a second hip fracture, especially in elderly patients. To lower the risk of a second hip fracture, any underlying causes for excessively using NSAIDs should be treated and thus fewer NSAIDs prescribed after a first hip fracture.

## Background

The occurrence of a second hip fracture and subsequent mortality in patients with a fragility hip fracture is high [1–6]. Ryg et al., using Denmark’s National Hospital Discharge Register for the period 1997 to 2001, explored this sequela of osteoporosis [2]. They found that patients with a fragility hip fracture had twice the risk of a second hip fracture, and that they had a 5-year mortality of approximately 60 %. Moreover, the estimated 1-year risk of a second hip fracture, whether in Western or Asian populations, should vary from 2 to 5 %, depending on the age of the patient [3]. These findings highlight the importance of formulating and proposing a tertiary strategy for osteoporosis to prevent a subsequent hip fracture [3, 7].

We previously reported that, in addition to age, female gender, and comorbidities, the prolonged use of analgesics, e.g., paracetamol, and anti-inflammatory medications, e.g., dexamethasone and NSAIDs (non-steroidal anti-inflammatory drugs), is a significant risk factor for a second hip fracture after hip fracture surgery [3]. Paracetamol, dexamethasone, and NSAIDs are commonly prescribed. Paracetamol is a mild analgesic and dexamethasone a potent anti-inflammatory corticosteroid. NSAIDs have analgesic effects and, in higher doses, anti-inflammatory effects because they inhibit the cyclooxygenase-2 (COX-2) activity and then reduce the synthesis of prostaglandins [8]. Prostaglandins are potent, multifunctional regulators of bone metabolism, which can stimulate and inhibit bone resorption and formation [9–13]. In general, NSAIDs are supposed to reduce the rate of bone loss, and thus improve bone mineral density (BMD) and prevent a fragility hip fracture [14–16], but conflicting results have also been published [17, 18]. Additional analyses that use different approaches to control for confounding are necessary to clarify the degree of association and/or causal link between NSAIDs and the risk of a second hip fracture.

During the last decade, interest in determining the degree of association and/or causal link between medications and the risk of a fragility hip fracture has grown [17, 19]. However, establishing the degree of association and/or causal link is difficult because observational studies are notoriously vulnerable to the effect of different types of confounding [20]. It is well recognized that the estimate of causality obtained by comparing cases with controls could be prejudiced because of problems such as selection bias or other systematic errors [20–22]. Rosenbaum and Rubin developed and popularized the propensity-score matching (PSM) method for observational (non-experimental) studies, in which only a small subset of controls comparable to cases must be selected [21]; PSM reduces the selection bias by balancing groups on the probability of being treated based on specific covariates [22]. Many studies have used the PSM method to control for confounding.

In the current study, we used the PSM method to control for confounding and aimed to determine the strength of the association between NSAIDs and the risk for a second hip fracture in patients after hip fracture surgery. We also explored the age-specific risk of a second hip fracture in these patients.

## Methods

### Data source

The Taiwan National Health Insurance (NHI) program, a single-payer universal program that began in March 1995, now enrolls more than 99 % of the population. Its claims data for reimbursements provide one of the world’s largest datasets for health research. The National Health Insurance Research Database (NHIRD), derived from the payment system of the National Health Insurance Administration (NHIA) and maintained by the National Health Research Institute (NHRI) in Taiwan, provides vital information for research. The accuracy of the NHIRD diagnoses of major disorders, e.g., stroke and acute coronary syndrome, has been validated [23]. The NHIRD includes patient demographics, disease diagnoses, medical care institution names, medical expenditure, and prescription claims data. For each medical expenditure reimbursement, the types of medical services and details of medical orders and costs are recorded. In the present study, data were obtained from the Longitudinal Health Insurance Database (LHID2000), a representative subset of 1 million patients from the NHIRD (http://nhird.nhri.org.tw/). All the identifiers of individual patients and medical care providers (medical professionals and institutions) are deleted by the NHIA before data are transferred to the NHIRD. Institutional review board (IRB) approval, an agreement to approve, monitor, and review biomedical and behavioral researches involving humans, is pre-approved by the NHRI for de-identified data.

### Study design and participants

**Fig. 1:**
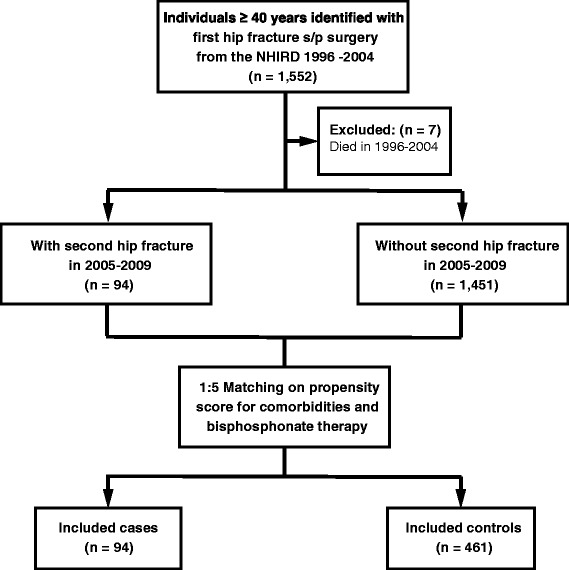
A flowchart of the patient selection process

This study was a nationwide population-based observational study using PSM to control valuables that are measured at baseline for confounding. The study participants were selected from the NHIRD/LHID2000 registry covering the period from January 1996 to December 2004 and followed longitudinally until December 31, 2009. The study participants were identified from the database based on the following criteria: (1) a diagnosis code of hip fracture (ICD-9-CM diagnosis codes 820.0–820.9); (2) a procedure code of internal fixation or partial hip replacement (ICD-9-CM procedure codes 79.15, 79.35, and 81.52); and (3) patients 40 years old or older. The first admission date for a hip fracture was defined as the index date. After deleting the records of patients who died between 1996 and 2004 (*n* = 7), 1,545 patients with a hip fracture were included and followed longitudinally until the end of the study. During the follow-up period, patients with a second hip fracture were selected as Cases (*n* = 94). Four hundred sixty-one of 1,451 patients were selected as Controls using 1:5 matching on propensity scores for comorbidities and bisphosphonate therapy. By matching variables that would otherwise confound comparisons between groups, the PSM method effectively creates similar case and control sets from an existing dataset for an observational study [22]. The Cases group consisted of 94 patients with a second hip fracture and the Controls group of 461 without. The flowchart of patient selection process is presented in Fig. 1.

### Drug exposure and primary and secondary endpoints

NSAIDs, identified based on the anatomical therapeutic chemical (ATC) classification system [24], are known to work in two ways: (1) analgesic effects at lower doses and (2) anti-inflammatory effects at higher doses. To clarify the dose-response relationship between NSAIDs and the risk of a second hip fracture, we included paracetamol (a pure analgesic) and dexamethasone (a corticosteroid and potent anti-inflammatory drug) in the current study. The prescription history of each patient for every medication studied was summarized as either exposed or not exposed during the paired case and control periods. For each patient, to explore the possible dose response, we also estimated the mean daily dose (MDD) by calculating the cumulative dose of medications prescribed and then divided that number by the number of days in the period. The primary endpoint of this study was to determine the strength of association between drug exposure and the occurrence of a second hip fracture in patients after hip fracture surgery. For our secondary endpoint, information on the age-specific risk of a second hip fracture in these patients was collected for further analysis.

### Statistical analysis

A *χ*^2^ test was used to analyze categorical data. For numerical variables, Student’s t test was used for between-group comparisons. Univariate and multivariate analyses using a stepwise logistic regression model were done to detect significant predictive factors of a second hip fracture after hip fracture surgery. Between-group comparisons were done by estimating the odds ratio (OR) and the 95 % confidence interval (CI) in a logistic regression model. To examine the effects of MDD on the risk of a second hip fracture, the Pearson correlation test was used. The 15-year second hip fracture-free survival rate after hip fracture surgery was estimated using the Kaplan-Meier method and the log-rank test. The cumulative hazard function was also used and the proportional hazards assumption was met for each parameter in additional models. In Cox-regression proportional hazards survival analysis, potential predictors in the forced-entry model were used as covariates (e.g., age, gender, and comorbidities). Significance was set at *p* < 0.05 (two-sided). SAS 9.2 (SAS, Inc., Cary, NC, USA) was used for all analyses.

## Results

From 1996 through 2004, 555 patients ≥40 years who sustained a hip fracture and underwent hip fracture surgery were selected and enrolled in this study. There were no significant differences in comorbidities and bisphosphonate therapy between the Cases and Controls (Table 1). Patients with a second hip fracture after hip fracture surgery were older (mean age: 74.0 years vs. 69.5 years, *P* < 0.001). There were, however, no significant differences in gender distribution.

**Table 1:** Characteristics of Cases and Controls by 1:5 matching on propensity score for comorbidities and bisphosphonate therapy

Variables	Cases (*n* = 94)	Controls (*n* = 461)	*P* Value
Comorbidities, no (%)			
Diabetes mellitus	34 (36.2)	147 (31.9)	0.420
Arterial hypertension	63 (67.0)	311 (67.5)	0.934
Hyperlipidemia	20 (21.3)	102 (22.1)	0.856
Coronary heart disease	34 (36.2)	158 (34.3)	0.725
Myocardial infarction	1 (1.1)	8 (1.7)	0.639
Cardiac dysrhythmia	18 (19.2)	71 (15.4)	0.367
PAOD^a^	3 (3.2)	21 (4.6)	0.554
Kidney dysfunction	2 (2.1)	4 (0.9)	0.282
Stroke/TIA^b^	4 (4.3)	26 (5.6)	0.589
Dementia	11 (11.7)	60 (13.0)	0.728
Parkinson’s disease	11 (11.7)	44 (9.5)	0.523
COPD^c^	24 (25.5)	113 (24.5)	0.834
Osteoporosis	25 (26.6)	133 (28.9)	0.659
Arthritis	33 (35.1)	145 (31.5)	0.489
Bisphosphonate therapy, no (%)	26 (27.7)	123 (26.7)	0.855
Propensity score, mean points (SD)	0.17 (0.04)	0.16 (0.03)	
(Min, Max)	(0.10, 0.43)	(0.08, 0.31)	
Age, mean years (SD)	74.0 (2.6)	69.5 (3.6)	<0.001*
Sex, no. (%)			0.995
Male	33 (35.1)	162 (35.1)	
Female	61 (64.9)	299 (64.9)	

^a^ PAOD, peripheral arterial occlusive disease; ^b^ TIA, transient ischemic attack; ^c^ COPD, chronic obstructive pulmonary disease

* *P* value < 0.05 is significant and all analysis was done by logistic regression model in SAS 9.2

**Table 2:** Medication use (paracetamol, dexamethasone, and NSAIDs) in Cases and Controls

Variables	Cases (*n* = 94)	Controls (*n* = 461)	*P* Value
Paracetamol, no. (%)	34 (35.8)	83 (18.1)	0.026*
MDD^a^, mg (SD)	605.6 (103.4)	496.0 (127.9)	0.009*
Aspirin, no. (%)	9 (9.9)	46 (10.0)	0.491
MDD, mg (SD)	105.6 (57.9)	101.2 (53.9)	0.569
Diclofenac, no. (%)	39 (41.7)	71 (15.4)	<0.001*
MDD, mg (SD)	206.9 (59.4)	116.1 (99.1)	<0.001*
Ibuprofen, no. (%)	26 (28.1)	93 (20.1)	<0.001*
MDD, mg (SD)	439.9 (115.9)	257.6 (85.0)	<0.001*
Naproxen, no. (%)	7 (7.4)	30 (6.5)	0.643
MDD, mg (SD)	485.2 (184.9)	451.0 (150.1)	0.583
Nabumetone, no. (%)	8 (9.0)	47 (10.1)	0.217
MDD, mg (SD)	1055.7 (479.3)	1014.7 (609.7)	0.506
Etodolac, no. (%)	13 (13.6)	54 (11.8)	0.416
MDD, mg (SD)	552.0 (50.1)	590.5 (33.9)	0.563
Celecoxib, no. (%)	22 (23.1)	44 (9.5)	<0.001*
MDD, mg (SD)	305.3 (98.8)	198.2 (100.1)	<0.001*
Rofecoxib, no. (%)	11 (11.6)	57 (12.3)	0.086
MDD, mg (SD)	70.6 (6.6)	75.7 (9.8)	0.057
Dexamethasone, no. (%)	37 (39.4)	76 (16.5)	<0.001*
MDD, mg (SD)	9.8 (7.5)	4.0 (3.9)	<0.001*

^a^ MDD, mean daily dose

* *P* value < 0.05 is significant and all analysis was done by logistic regression model in SAS 9.2

**Fig. 2:**
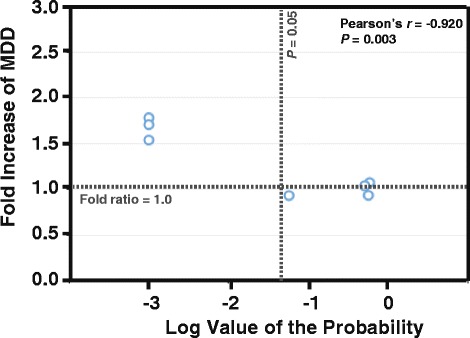
Correlation between the fold increase of the mean daily dose (MDD) of NSAIDs and of the log value of the probability of the current statistical hypothesis tests

Patients in the Cases group had taken significantly more paracetamol and had a higher MDD than did those in the Controls group (35.8 % vs. 18.1 %, *P* = 0.026; 605.6 mg vs. 496.0 mg, *P* = 0.009, respectively); the same was true for diclofenac (41.7 % vs. 15.4 %, *P* < 0.001; 206.9 mg vs. 116.1 mg, *P* < 0.001), ibuprofen (28.1 % vs. 20.1 %, *P* < 0.001; 439.9 mg vs. 257.6 mg, *P* < 0.001), celecoxib (23.1 % vs. 9.5 %, *P* < 0.001; 305.3 mg vs. 198.2 mg, *P* < 0.001), and dexamethasone (39.4 % vs. 16.5 %, *P* < 0.001; 9.8 mg vs. 4.0 mg, *P* < 0.001). There were, however, no significant between-group differences for aspirin, naproxen, nabumetone, etodolac, or rofecoxib (all *P* ≥ 0.057) (Table 2). There was a highly negative correlation between the MDD ratios and the probability values of the current statistical tests (Pearson’s *r* = −0.920, *P* = 0.003 for NSAIDs only) (Fig. 2); thus, the higher the MDD ratios were, the greater risks of a second hip fracture the patients had.

Using the PSM method to control for confounding, age but not gender was identified as a significant predictor of a second hip fracture (Table 1). For every 1-year increase in age, there was at least a 2.4 % increase in the risk of a second hip fracture, and at least a 13.0 % increase for every 5-year increase in age (all *P* ≤ 0.044) (Table 3). Overall, the risk ratio (RR) was 14.8 % higher for female patients than for male patients (*P* < 0.001) (Table 4). Using 40–49-year-olds as the reference group, the RRs for a second hip fracture were significantly higher for those 60–69, 70–79, and ≥80 (all *P* ≤ 0.040), but not for those 50–59 (*P* = 0.627).

**Table 3:** Odds ratio estimates in logistic regression model for second hip fracture in patients after hip fracture surgery

Effect	Point Estimate	95 % Wald Confidence Interval		*P* Value
		Lower	Upper	
One-year age difference				
Total vs. total	1.026	1.004	1.047	0.017*
Female vs. female	1.024	1.001	1.050	0.044*
Male vs. male	1.030	1.002	1.059	0.038*
Female vs. male	1.190	1.005	1.214	<0.001*
Five-year age difference				
Total vs. total	1.134	1.022	1.259	<0.001*
Female vs. female	1.130	1.010	1.278	<0.001*
Male vs. male	1.162	1.013	1.333	<0.001*
Female vs. male	1.220	1.010	1.430	<0.001*

* *P* value < 0.05 is significant and all analysis was done by logistic regression model in SAS 9.2

**Table 4:** Analysis of parameter estimates in lognormal regression model for second hip fracture in patients after hip fracture surgery

	Parameters	Estimate	SE	95 % Wald CI		Chi-	Pr >	RR
				Lower	Upper	Square	ChiSq	
Sex	Male	-	-	-	-	-	-	1.000
	Female	1.001	0.342	0.113	1.772	2.80	<0.001*	1.148
Age	40–49	-	-	-	-	-	-	1.000
	50–59	−0.401	0.488	−1.357	0.556	0.85	0.627	0.670
	60–69	0.756	0.357	0.057	1.455	3.55	0.040*	2.129
	70–79	0.820	0.255	0.319	1.320	4.05	0.020*	2.270
	≥80	1.013	0.265	0.494	1.531	8.87	0.005*	2.752

* *P* value < 0.05 is significant and all analysis was done by logistic regression model in SAS 9.2

**Fig. 3:**
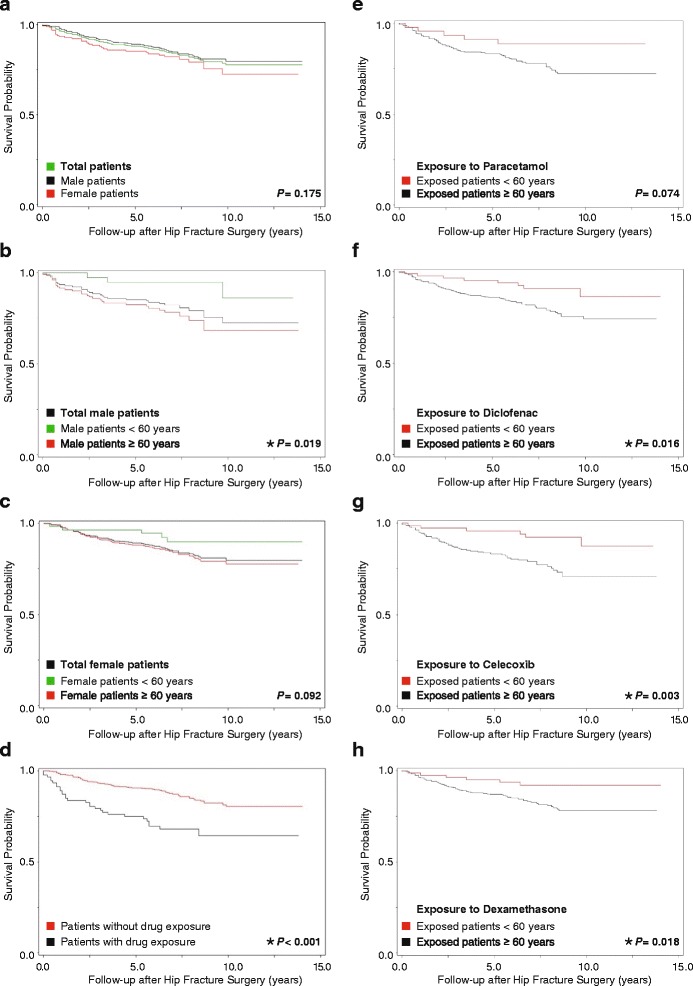
Kaplan-Meier survival estimates with a second hip fracture (SHFx) as an endpoint. Survival free of a second hip fracture for **a** total patients, **b** male patients, **c** female patients, **d** patients who took and did not take paracetamol, NSAIDs, and dexamethasone, and **e** patients who took paracetamol, **f** diclofenac, **g** celecoxib, and **h** dexamethasone. * *P* < 0.05

The 15-year survival analyses showed that male patients ≥60 were significantly more likely to have a second hip fracture after their hip fracture surgery than those <60 (*P* = 0.019). This was not true for the total group of patients or for the group of female patients (both *P* ≥ 0.092) (Fig. 3a–c). For the group as a whole, the RR for a second hip fracture was significantly (*P* < 0.001) and time-dependently higher for patients taking paracetamol, NSAIDs, and dexamethasone (Fig. 3d). Kaplan-Meier survival analyses also showed that the RR for a second hip fracture was significantly higher for patients ≥60 than for those <60 exposed to diclofenac (*P* = 0.016) (Fig. 3e), celecoxib (*P* = 0.003) (Fig. 3f), and dexamethasone (*P* = 0.018) (Fig. 3g), but not to paracetamol (*P* = 0.074) (Fig. 3h).

## Discussion

We confirmed that patients who take NSAIDs after hip fracture surgery have a significantly higher risk for a second hip fracture than do patients who do not take NSAIDs. Moreover, the positive association was dose- and time (years after first hip fracture surgery)-dependent for the group as a whole and dose- and age-dependent for patients ≥60. Furthermore, the higher the MDD of NSAIDs was, the greater the risk of a second hip fracture was. These findings highlight the importance of changing the treatment strategy of elderly patients after their first hip fracture.

To the best of our knowledge, this is the first report on confirming the association between taking NSAIDs and the risk of a second hip fracture in patients after hip fracture surgery. We previously reported that age, female gender, comorbidities, and the prolonged use of analgesics and anti-inflammatory medications are all significant risk factors for a second hip fracture after hip fracture surgery, but that bisphosphonate therapy was protective [3]. However, in an observational study, there are many confounders that usually distort the relationship between an exposure and an outcome [20–22, 25]. The PSM method is useful in these circumstances because it provides a neutral weighting formula that yields unbiased estimates of the effects of treatment. In the current study, we thus used the propensity score model for controlling variables that are measured at baseline. After using the PSM for controlling comorbidity and bisphosphonate therapy, age but not gender remains to be a significant risk factor for the occurrence of a second hip fracture in patients after hip fracture surgery.

Although users of NSAIDs are reported to have higher BMD than do non-users [19, 26, 27], the effect of NSAIDs on the risk of a hip fracture is still not understood [14–18]. Two studies found that the hip fracture risk was lower in patients taking NSAIDs [14, 17]. In contrast, a report based on the Danish Osteoporosis Prevention Study (DOPS) showed that users of NSAIDs had more hip fractures than expected [18]. Leaving aside the debate whether NSAIDs increase the risk of a first hip fracture, the authors highlight the issue of tertiary prevention for osteoporosis because of the healthcare resources constraint [2, 3]. Our data revealed that the higher the MDD ratios of NSAIDs, the greater the likelihood of a second hip fracture in patients after hip fracture surgery. One possible explanation is that, when prolonged, the anti-inflammatory effects of NSAIDs at higher doses elevate the risks of a second hip fracture. Another explanation is that NSAID users have an impaired gait because of the sequelae of their index fragility hip fracture. No matter what the mechanism is, we recommend that NSAIDs should be cautiously prescribed for patients who have just undergone hip fracture surgery, particularly for elderly patients. As with other drugs prescribed for elderly patients [17, 28, 29], the most judicious approach is to restrict NSAIDs to situations in which their benefits clearly overweigh their risks, and to use them only after any underlying causes for excessive drug use have been adequately treated and potentially safer alternatives have been tried. When treatment with NSAIDs is necessary, the lowest feasible dose should be used for the shortest duration required to achieve the desired effect [28, 29].

This study has some limitations. First, it is retrospective. Second, it might still have an uncontrolled selection and recall bias despite our using stratification and the PSM method to control for confounders. For example, the presence of a diagnosis of comorbidity is different from controlling for the severity of the disease, which may be expected to be different in older individuals. Third, the risk of a second hip fracture may be underestimated because we included the survival rate after the index hip fracture only and also excluded patients who died from any cause between 1996 and 2004. We could not account for patients who sustained a hip fracture 2 years before the index date and thus wrongly had their second hip fracture classified as an index hip fracture despite their having no hips at risk. Finally, we lacked information on what caused the higher exposure rate and higher MDD of NSAIDs in our patients after their hip fracture surgery.

## Conclusions

In conclusion, taking NSAIDs after hip fracture surgery significantly increases the risk of a second hip fracture. The positive association is dose- and time-dependent for all patients and age-dependent for the elderly. Because of a global deterioration of health conditions in elderly patients after a fragility hip fracture [17], they are usually prescribed significantly more drugs, especially analgesics and anti-inflammatory agents. Physicians should limit prescribing NSAIDs to situations with a positive benefit-risk balance and use them only after the underlying cause for excessively using NSAIDs have been adequately treated and potentially safer alternatives have failed. The lowest feasible dose for the shortest duration required to achieve the desired effect should be considered. If more long-range treatment with NSAIDs is necessary and inevitable, more aggressive monitoring and prevention for another fragility fracture is warranted [30, 31], because long-term NSAIDs treatment might contribute to rises in the risk for a second hip fracture.

### Ethic approval or consent to participate

Not applicable.

### Consent for publication

Not applicable.

### Availability of data and materials

The website of the Taiwan National Health Insurance Research Database is found at http://nhird.nhri.org.tw/.

## Abbreviations

ATC: anatomical therapeutic Chemical
BMD: bone mineral density
CI: confidence interval
COX-2: cyclooxygenase-2
DOPS: Danish Osteoporosis Prevention Study
ICD-9-CM: 9th revision of International Classification of Diseases, Clinical Modification
IRB: Institutional review board
LHID: Longitudinal Health Insurance Database
MDD: Mean Daily-Dose
NHI: National Health Insurance
NHIA: National Health Insurance Administration
NHIRD: National Health Insurance Resarch Database
NSAIDs: Non-Steroidal Anti-Inflammatory Drugs
OR: odds ratio
PSM: propensity-score matching
RR: risk ratio
